# Inhibitors and facilitators to the utilization of postpartum care in China: an integrative review

**DOI:** 10.1186/s13690-022-01000-3

**Published:** 2022-12-06

**Authors:** Xiaoqian Zhang, Catharina Matheï, Mieke Vermandere, Xiaoli Zuo, Qian Wang, Hui Leng, Tang Li, Frank Buntinx

**Affiliations:** 1 Academic Center for General Practice, Catholic University of Leuven, Leuven, Belgium; 2 Qingdao United Family Hospital, Qingdao, China; 3 Department Translational Research in Gastrointestinal Disorders, Catholic University of Leuven, Leuven, Belgium; 4 The Affiliated Hospital of Medical College Qingdao University, Qingdao, China

**Keywords:** Postpartum care, China, Influencing components, Inhibitors, Facilitators

## Abstract

**Background:**

Postpartum care is an expanding concept in China, and it is gaining vast attention in Chinese society. However, due to some Chinese traditions and rituals during the postpartum period, the utilization of modern postpartum care should be improved on both individual and community levels from different aspects. This integrative review outlined the inhibitors and facilitators of postpartum care utilization in China.

**Methods:**

Writing an integrative review, a literature search was conducted in Chinese and English databases including Wan Fang, China National Knowledge infrastructure, Medline, Web of Science, and Embase till 31 October 2021 to capture citations covering ‘postpartum care’, ‘utilization’ and ‘China’. Titles and abstracts were screened independently by three reviewers. Included studies were critically appraised using tools and checklists independently for both qualitative and quantitative studies by two different reviewers who also performed thematic synthesis.

**Results:**

Of the 4359 citations screened, 41 studies (450,788 patients) were selected. Categorization of the factors influencing postpartum care utilization revealed five components: sociocultural (25 studies); educational (24 studies); organizational (12 studies); economic (19 studies); and physical (6 studies). Factors influencing postpartum care utilization both on individual and community levels were identified. They included facilitated factors such as higher mother’s and partner’s education level, higher socioeconomic status, lower parity, better insurance coverage, urban geographical location, Han ethnicity, and better transportation. Inhibitory factors such as under-managed policy regulation, migrants without domicile, and lower quality of care were also reported.

**Conclusion:**

This review has identified the inhibitors and facilitators of postpartum care utilization in China. Five major aspects including sociocultural, educational, organizational, economic, and physical components have been analysed. Results can be used to improve the utilization of modern postpartum care on both individual and community levels in Chinese society.

**Supplementary Information:**

The online version contains supplementary material available at 10.1186/s13690-022-01000-3.

## Introduction

The postpartum period – a time for individual and family transition involving physical, social, and mental recovery after pregnancy and giving birth – is crucial for women’s body to restore changes related to pregnancy within a few weeks after birth. The maternal mortality rate during this period can even be higher than that during pregnancy and giving birth [1], which might constitute a direct impact on the mental and physical health of mothers and new-borns, and even their family members. It is important to build a comprehensive care plan for both mother and offspring, including the whole family, according to the woman’s individual desires and needs. Ongoing care, rather than a single encounter, is required to optimize the postpartum health of mothers and their families.

The regions in China have different guidelines for postpartum care (PPC). Compared to other countries, the overall quality of Chinese postnatal care guidelines is unsatisfied [2]. In general, the average hospital stay for uncomplicated births was usually approximately 5 days [3]. The first PPC visit is commonly recommended within one week after birth, and the second time is around six weeks postpartum [4]. Both home visit and consultation in health facilities are recommended by different health workers within six to eight weeks after delivery [3]. Primary, secondary, even tertiary hospitals are chosen according to patient’s preference. The recommended postpartum care content includes physical examinations, additional laboratory check-ups, general health education, nutritional guidance, breastfeeding counselling, mental problem screening, family planning counselling, etc. [5]. In China, a unique PPC program suited for local situations is necessary in light of its own traditions and rituals. The overall postpartum follow-up rate for mothers in China has been improving in recent years, from 46% in 1993 to 75% in 2018, including home visit and consultation [6]. The Chinese Health System Reform, initiated in 2009, played an important role in the PPC service, as it became one of the top priorities of essential public health services. The reform advocates equality; however, regional PPC disparities remain remarkable in China [7].

Given the above background, an evidence synthesis is required to analyse the different promotors and inhibitors of PPC service utilization in China. Our review focused on synthesizing evidence concerning barriers/facilitators in the maternal aspect of PPC. Through an initial search process, reviews were found to present factors influencing PPC utilization in different countries with diverse methodologies [8, 9]. Only limited data about China was presented in these reviews as none of them included studies in Chinese. Reviews of English-only literature led to an incomplete information synthesis. Therefore, we conducted a comprehensive evidence synthesis based on the literature available in both English and Chinese to outline the factors associated with PPC utilization in China.

## Material and methods

The integrative review used the framework outlined by Whittemore et al. [10] and Phillips et al. [11], which initially involved a clear identification of the aim of the review. Afterwards, a well-defined literature search was conducted in English and Chinese language to ensure all related literature is included. The further data evaluation stage involved comparing and analysing the similarities and differences in findings from each publication after the quality check. Similar concepts were compared and grouped intentionally into different components inductively and deductively, with inspiration from the referenced article where sociocultural, educational, organizational, economic, and physical components were mentioned [9]. At the final stage, the components were presented to reveal the process of data synthesis and integration. Ethical approval was not required for an integrative literature review.

### Literature search and selection

Literature searches were conducted from database inception until the end of October 2021 in three English databases: Medline, Web of science, Embase, and two Chinese databases: Wan Fang, China National Knowledge Infrastructure. We used combinations of the descriptors ‘postpartum care’, ‘China’, and ‘utilization’ in both English and Chinese languages, applying all their synonyms and associated word variants, searching title, abstract, keywords, and the Mesh Terms (see supplementary file, Table A). The Chinese core journal lists (the journal lists of Chinese Social Sciences Citation Index, Chinese Science Citation Database, and a Guide to the core journals of China) have been widely recognized by the Chinese academic community as high-quality peer-reviewed journal lists [12]. Publications that were included both in the Chinese core journal lists and the two Chinese databases were chosen during the search process to ensure data quality. Relevant references identified during the reading of already selected publications were also included as part of the search results.

Identified publications were selected for further analysis, based on the following inclusion criteria: (1) specific reference to facilitators and inhibitors of utilization of postpartum care service in China; (2) published either in English or Chinese; (3) available in the original full text. Articles, books, and dissertations were all included. No restriction was placed on the type of the study methodology. We excluded studies of postpartum periods not organized in China, those of antenatal and prenatal care, and other grey literatures rather than dissertations and books, such as government reports, posters or infographics which have not been peer reviewed. Three primary reviewers (XQ.Z, XL.Z, Q.W) did twice title and abstract selecting and prepared a provisional list of articles after duplicates were removed. Then, further assessment for eligibility based on the inclusion and exclusion criteria was held followed by the three primary reviewers.

### Critical appraisal

We used the Critical Appraisal Skills Programme (CASP) Qualitative Research Checklist [37] to evaluate the quality of qualitative studies, the Agency for Healthcare Research and Quality (AHRQ) checklist [38] for assessing the cross-sectional studies, the Mixed Methods Appraisal Tool (MMAT) for mixed-methods studies, and the Joanna Briggs Institute (JBI) Critical Appraisal tools for quasi-experimental studies [39]. The cataloguing and systemizing of the evidence were performed by the three primary reviewers. The final interpretations were carried out together by three senior authors as reported in the supplementary file (C.M, M.V, F.B).

### Evidence synthesis

Publications which were included in this integrative review were read by the three primary reviewers several times independently to make a sense of the whole. Texts which include factors influencing the utilization of PPC in China were systematically coded for data reduction. The similarities and differences between the coded texts were compared to determine which codes could be grouped together. The codes were grouped into different sub-concepts by combining the codes that were related to each other. Finally, the sub-concepts were compared and grouped under the five components (sociocultural, educational, organizational, economic, and physical components).

## Results

### Study selection

**Fig. 1:**
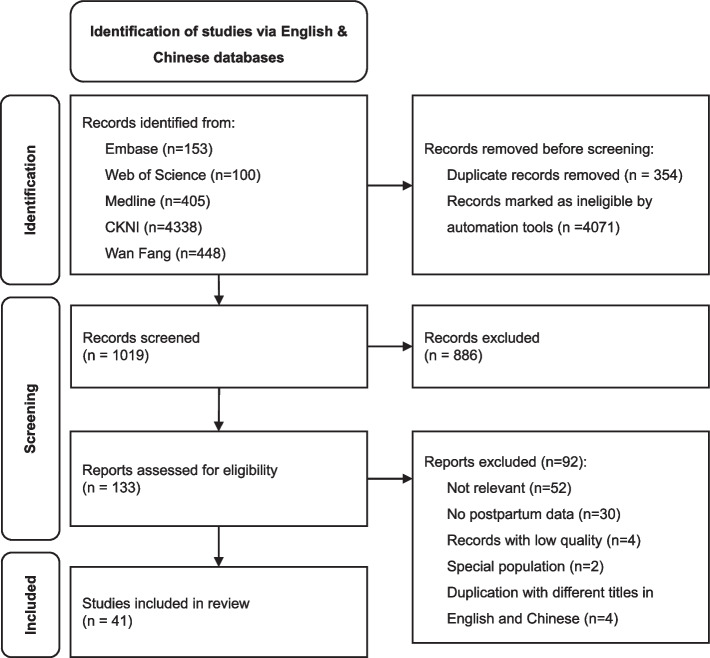
Flowchart for relevant literature in English and Chinese databases

We found 545 articles published from the Chinese core journal list by the end of October 2021 in Chinese databases. Sixty-six articles remained after removing duplications and title selection. After assessment of full text for eligibility and critical appraisal, 13 Chinese articles were included for synthesis. No additional referencing data followed cross-referencing (snow balling technique) was added (Fig. 1). In the three English language databases, 558 published articles were found. After removing duplications, 67 articles were chosen based on title and abstract selection. Rayyan.ai was used during the first title selection procedure. Unrelated titles and abstracts will be automatically rated by this ai system. The ai system ranked the articles as high related if they contain pre-defined words such as ‘China’, ‘postpartum’, ‘care’, etc. Two additional publications from snow balling were added. Twenty-eight English articles were finally embraced after assessment for eligibility and critical appraisal (Fig. 1).

Of the 41 articles included in this review, there were 33 cross-sectional studies, three mixed methods research projects, two qualitative research projects, two randomized controlled trials and one quasi-experimental study. Most of the studies were published in the last decade. Five articles were found both in Chinese and English database with bilingual abstract [15, 16, 40–42]. We categorized these five articles under the English data. There are two articles which were written by the same author with the same original data and published in different years (2010 and 2015) in English and Chinese [43, 44]. Only the English version was included in the result because this version allowed for better in-depth analysis, and the methodology was better described in detail.

### Description of studies

**Fig. 2:**
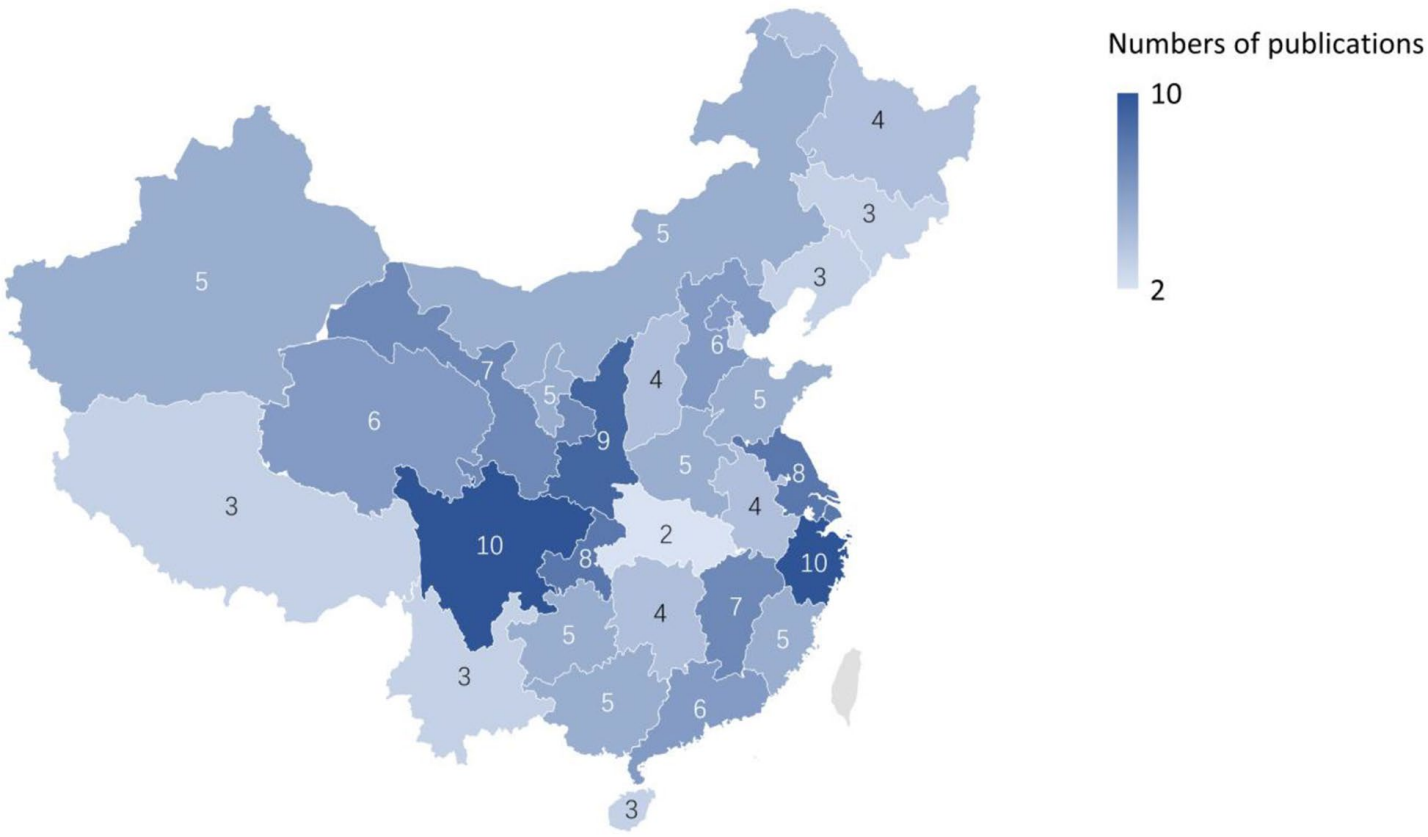
Numbers of publications in different districts in China

Three studies were written by English authors, the rest of the authors were from Chinese origin. Of these 41 articles, several studies have commented about multiple provinces. There were 17 studies that commented about the situation in western China, 19 about eastern China, 11 about central China, and 3 about the whole China (Fig. 2). The study size ranged from less than hundred to more than 300,000 participants, and the postpartum service utilization ratio was reported as lower than 10% to 99%. We noticed 92% of Chinese data was focused on cities, while 86% of the English data was focused in rural area.

### Data synthesis

Categorization of the factors influencing PPC utilization revealed five components: sociocultural (25 studies); educational (24 studies); organizational (12 studies); economic (19 studies); and physical (6 studies). The sociocultural component incorporated a wide range of individual and community factors. The educational component included elements of the educational background of both mothers and families, and skills or responsibilities of the medical staff. The organizational component referred to different programs and services in quality care and governmental policies. The economic component involved insurance, financial, and incentive issues with their family, community, and society aspects. Finally, the physical component aims to describe the influence from distance and transportation factors. These five components were further processed into two sub-levels, namely the individual and community level. The individual level refers to determinants of a personal and familial scope; while the community level assigns to determinants of institution, insurance, society, and healthcare system.

### Major components

#### Sociocultural component

The inhibitors and facilitators for sociocultural component was divided into two sub-levels: an individual and a community level, including social economic status, cultural beliefs, legality of the birth, ethnical situation, housing condition, reproductive characteristics, and geographical influences. At individual level, the most common inhibitor was disadvantages in subsistence for mothers. These included women with more children, unemployment, older maternal age, older husband’s age, low socioeconomic status, conservative mentality, and no registered domicile [13–15, 17–20, 22–33, 41, 45]. Young (< 20 years old) or advanced (> 30 ~ 40 years old) maternal age were associated with less postnatal service utilization [15–17, 24, 25, 30, 32]. Domiciled resident, non-agricultural hukou (a household registration under the supervision of the Ministry of Public Security of P. R. China, categorized into agricultural and non-agricultural types), and a partner with a local hukou were positively associated with postnatal service usage [20, 22–26]. One article has shown that the older the husband’s age (above 30 ~ 35 years old), the lower the utilization ratio of postnatal care, while husband’s employment status didn’t significantly influence the use of postnatal care [18]. Women who delivered her first child or had a child with low birth weight have a higher PPC utilization rate [16, 24–26, 28–31]. We classified these reproductive characteristics under the socio-cultural facilitated determinants because these characteristics focused only on mother’s individual situation in our selected studies. A better socioeconomic status, women with an occupation, and certain occupation types (white collar or non-farmer) facilitated postnatal period care use [17, 26–28]. Only one study has shown the opposite result that maternal unemployment can positively affect the PPC usage [14]. At community level, one-child policy was considered as an important influencing factor to postnatal care services, but the influence is controversial [14, 21, 25]. Ethnic minority women in rural China had less postnatal care utilization than Han ethnicity [27, 30]. A facilitator at the community level was urban (versus rural) geographical allocation with easy access to maternal health resources [31, 34–36] (Table 1).

**Table 1:** Inhibitors and facilitators for the socio-cultural component

**Inhibitors**		**Facilitators**	
**Individual level**	**Community level**	**Individual level**	**Community level**
**Cultural** • Saving face culture/feels shameful when seeking help [13] **Domicile** • Migrants without domicile [14] **Socioeconomic status** • Maternal occupation [14] **Reproductive characteristics** • Multipara [15–19] • Older husband’s age [18] • Older maternal age [20]	**Legality** • Unauthorised birth [21] **Cultural** • Traditional Chinese confinement [13]	**Domicile** • Domiciled resident [20, 22, 23] • Non-agricultural hukou/occupation [24, 25] • Partner is a local resident [26] **Socioeconomic status** • Socioeconomic status [27] • Maternal occupation [17, 26, 28] • Living with parents in-law [24] **Reproductive characteristics** • First child [24–26, 28–30] • Low birth weight [31] • Young maternal age [24, 30, 32] • Advanced maternal age [15–17, 25] • Vaginal delivery [32] • Giving birth in hospital [33]	**Geographical influence** • Geographical allocation (urban vs. rural) [18, 31, 34–36] **Legality** • Violated the one-child policy [14] • Authorized birth [25] **Ethnics** • Han ethnicity [27, 30]

#### Educational component

The inhibitors and facilitators of the educational component were divided into two sub-levels (individual and community levels) and further into four domains: education, information sharing, knowledge & skills & responsibility, and training. The barriers at an individual level were the lack of awareness of maternal postnatal health care and poor awareness of self-health care [16, 46]. At a community level, the most well-known inhibitors were poor educational background, limited skills, and limited responsibility of health providers [3, 13, 47]. Most studies have shown a positive relationship between maternal or partner’s education level and utilization rate of postpartum care [4, 17, 20, 22, 24–30, 36, 41, 48, 49]. Certain training intervention to healthcare workers has shown positive effect to the utilization of PPC [5] (Table 2).

**Table 2:** Inhibitors and facilitators for the educational component

**Inhibitors**		**Facilitators**	
**Individual level**	**Community level**	**Individual level**	**Community level**
**Education** • Partner's educational level [14] • Consider medical problem as a stigma [13] **Information sharing** • Lack of awareness on postpartum care [16, 46]	**Knowledge, skills & responsibility** • Poor educational background, limited skills/knowledge/inadequate training of health providers [3, 13, 47, 50] • Limited responsibility of health providers / uncaring manners / little consideration of potential health problems [13]	**Education** • High maternal education level [4, 19, 20, 22, 24–30, 36, 41, 48, 49] • Partner's education level [17, 26, 48]	**Training** • Training intervention to healthcare workers [5]

#### Organizational component

Under the organizational component, six domains emerged under the facilitators and inhibitors: place of births, staffing, contact with health workers, policies, quality of postpartum care, and logistics. Giving birth in a high-level hospital (secondary and tertiary hospital) can negatively affect the follow up plan, while giving birth in primary or lower medical institutions influenced the PPC positively [4, 28]. Understaffing of healthcare workers and insufficient equipment and techniques in healthcare facilities might increase the dissatisfaction of patients and inhibited PPC utilization [13, 30, 47]. Prenatal care attendance was a strong predictor that related to a high rate of postnatal services utilization [30, 33]. Three studies have shown the important influence of local policies [4, 51, 52]. One study has shown the major improvement after the Chinese healthcare reform, because this reform from 2009 has appended the postnatal visits as part of the essential public health service [4]. A systematic follow-up schedule initiated by medical staff, regular home visits, and high quality of PPC were facilitators [3, 13, 31] (Table 3).

**Table 3:** Inhibitors and facilitators for the organizational component

**Inhibitors**	**Facilitators**	
**Community level**	**Individual level**	**Community level**
**Place of births** • Gave birth in secondary and tertiary hospitals vs. in ≤ primary institution [4, 28] **Staffing** • Understaffing of healthcare workers [3, 47, 50]	**Contact with health workers** • Prenatal / antenatal care attendance [30, 33]	**Policies** • Healthcare reform / Compensation policy adjustment [4, 51, 52] **Quality of postpartum care** • The quality of postpartum care [3, 13, 31] **Logistics** • Well-equipped facilities [13, 30]

#### Economic component

Under the economic component, low family income, and self-paid medical expenses were important inhibiting individual issues for delivering PPC services to Chinese women and vice versa [13, 15, 17, 26, 28, 33, 36, 41, 48, 49, 53], especially in floating populations. Chinese floating population assigns to persons staying away from their place of registration without having transferred their hukou. At community level, one Chinese qualitative study has shown negative effects from inadequate governmental investment in maternal and child health care, such as unguaranteed salary for medical staff [50]. Obtaining medical cost coverage, including the new cooperative medical insurance scheme, poverty alleviation insurance, commercial insurance, or coverage from employers or governmental compensations, can positively affect the final PPC utilization [14, 19, 26, 31, 41, 48, 54]. Limited facilitate influence from free postnatal consultations was noticed in a poverty population [40] (Table 4).

**Table 4:** Inhibitors and facilitators for economic component

**Inhibitors**		**Facilitators**	
**Individual level**	**Community level**	**Individual level**	**Community level**
**Financial** • Out-of-pocket medical expenses [13]	**Resources for health care providers** • Inadequate governmental investment in maternal and child health care caused resulting in unguaranteed salaries for health care workers [50]	**Financial** Family/client’s income, better PPC utilization [15, 17, 19, 26, 28, 33, 36, 41, 48, 49, 53]	**Insurance** • Covered by medical insurance [14, 41, 54] • Compensation for giving birth fee expenses by employers or government [19, 26, 31, 48] **Payment methods** • Free postnatal consultation for poverty population [40] • Conditional cash transfer program [55]

#### Physical component

Three studies have clearly shown the relationship between the distance to the nearest health care facility and the utilization ratio [24, 28, 47]. The longer the time needed to travel to the township health centres, the lower the rate of postpartum visits among women. At a community level, inconvenient transportation for clients due to inadequate geographical location inhibited PPC service utilization [47, 50]. Different geographical divisions (western, eastern, and north China) had different utilization of postpartum visits [17, 43] (Table 5).

**Table 5:** Inhibitors and facilitators for physical component

**Inhibitors**	**Facilitators**	
**Community level**	**Individual level**	**Community level**
**Transportation/accessibility** • Inconvenient transportation caused by the geographical environment [47, 50] **Geographical influence** • Different geographical/regional divisions [17, 43]		**Living area** • Urban area has higher postpartum visit rate than rural area [43]
	**Distance** • The closer the distance to the health facilities, the more postpartum visits [24, 28, 47]	

## Discussion

### General results

This integrative review synthesized the inhibitors and facilitators of PPC utilization in China from 41 publications into five components, including socio-cultural, educational, organizational, economic, and physical components.

Due to the sociocultural evolvement of the Chinese society in recent decades, millions of Chinese workers were considered as inter-provincial migrants with high population mobility. Four Chinese studies have shown the household registration system called Hukou negatively impacted on PPC utilization to internal migration [22, 23, 25, 26]. The medical insurance can’t be transferred in time due to the inability of the Hukou transfer. This coverage shortage from insurance system can further affect the PPC utilization negatively [14, 41, 54]. As greater attention has been paid by authorities to internal migrants, the negative influence from Hukou to PPC utilization has become milder. Chinese health system reforms focus on PPC rate because an important target in the standard care manual [6] has helped to steer utilization in the positive direction.

The saving face culture was one of the most important sociocultural barriers, followed by poor awareness of self-healthcare [16, 46] that affected the utilization of PPC. With the profound Chinese traditions dating back to the ancient Chinese history, the conservative thoughts and traditional Chinese saving face culture prohibited women to go for routine PPC check-ups. This culture barrier existed also in other countries, especially in East Asia [56, 57]. To overcome this, systematic approaches from organizational and educational domains were needed. Promotion of PPC services should already start during the pregnancy. Studies have highlighted the facilitating effects of prenatal attendance to improve the PPC utilization ratio [30, 33]. An appropriate training system for health care providers in primary level, adequate financial support in certain areas, and sufficient physician number per citizens are considered to be the requisite for improvement. However, there was still a serious shortage of qualified general practitioners in China. A continuing and high-quality systematic training program with an appropriate evaluation process would improve the current situation. Furthermore, health workers’ motivation was one of the impact factors to the PPC utilization, due to the fact that the home visit is planned in some regions automatically by the health workers, others were arranged according to patients’ requests [50]. From the governmental level, policies with a supporting insurance system, guaranteed salaries system for health care workers, proper funding, an organized health service management with coordination, and acquired human resources should be considered.

Compared to the other countries, Chinese medical system was originally introduced from the Soviet Union’s specialist-care medical model [58]. Under the organizational component of this integrative analysis, studies have shown Chinese people prefer to attend high-levelled healthcare facilities with better equipment and high quality of PPC service [3, 13, 30, 31, 47]. However, giving birth in a high-level hospital (secondary and tertiary hospital) can negatively affect PPC utilization [4, 28]. This was mainly caused by low qualified primary healthcare centres and insufficient information sharing between different medical settings as reported in other studies [8, 59, 60]. Barrier-free information sharing between different facilities or settings, regulation and optimizing of the primary care would improve the current situation. A previous study has also shown similar negative influence arising from lack of care continuity and lack of trust in providers of PPC [61].

Other barriers to PPC utilization in China have been identified by this integrative analysis under economic and physical components, including an inconvenient family financial status, an inadequate insurance coverage, and a lack of physical access. These barriers were also proved in other studies in which comparable inhibited factors to PPC were mentioned [62–65]. Therefore, the study emphasized the importance of promoting home-based care services for mothers and babies, with additional insurance coverage or compensation from governmental level.

Furthermore, there were major differences between different regions in the same province. Another study has also shown significant influences from regional differences on postpartum care utilization [66]. Through this integrative review, controversial findings were noticed in some different components. Certain studies have shown strong correlation between family income and PPC utilization, while other studies have shown no correlation [20, 54]. Two studies have identified that maternal employment has no influence on PPC utilization [20, 48], while other studies have identified facilitating effects [17, 26, 28] or inhibiting effects [14]. Two studies in the Jiangsu province written by the same author even reported contrary results [14, 28]. First of all, the data collection was different between these two studies that one was collected from national surveys, while the other used self-designed questionnaires. As the Chinese maternal health care system was regulated by the national medical bureau but implemented through the municipal administrations, this would cause a big regional difference regarding PPC utilization ratio and influencing factors. The relatively weak control from the national medical bureau for PPC services allowed regional administration to have more flexibility in the policy-making processes [43]. To overcome this, stronger administrative support from national medical bureau would be beneficial to overcome the regional influences. Additional nationwide studies with comparative Chinese regional data might evaluate reasons for the differences in PPC utilization to inform practice and policy.

### Strengths and limitations of the review

This review integrated publications with diverse methodologies, and includes both Chinese and English publications, using recommended integrative review approaches [10, 11]. Differences between English and Chinese literatures were analysed, dividing the information into five components, and analysing at both individual and community levels. By using it, different subjects were able to be classified based on the values of a set of predictor variables. These aspects of the review facilitated the evidence synthesis for drawing valid conclusions.

Most of the publications we selected were focused on small population groups, stratified by areas or ethnicities, only a limited number of publications were based on national data. No further comparison was made for other countries in this integrative review.

## Conclusion

Postpartum care utilization is expanding dramatically among the Chinese society, while some Chinese traditions and rituals prohibits the Modern postpartum care services. This integrative review outlined the inhibitors and facilitators of the postpartum care utilization in China on five major influencing aspects including sociocultural, educational, organizational, economic, and physical components. The results can be used to improve the postpartum care services among the Chinese society with both the individual and community level measures.

## Supplementary Information

**Additional file 1: Supplementary Material.** Studies relating to the influencing factors for postpartum utilization in China.

## Data Availability

Datasets used or analyzed during the current study are available from the corresponding author upon reasonable request.

## Abbreviations

PPC: Postpartum care
CASP: Critical Appraisal Skills Programme
AHRQ: Agency for Healthcare Research and Quality
MMAT: Mixed Methods Appraisal Tool
JBI: Joanna Briggs Institute
