# Supplementary material for: Inhibitors and facilitators to the utilization of postpartum care in China: an integrative review
Source: Arch Public Health. 2022 Dec 6;80:247. doi: 10.1186/s13690-022-01000-3 (PMC9724330; doi:10.1186/s13690-022-01000-3)
Supplement: Supplementary file 1 — Additional file 1: Supplementary Material. Studies relating to the influencing factors for postpartum utilization in China. [file 13690_2022_1000_MOESM1_ESM.docx]

Supplementary Material: Studies relating to the influencing factors for postpartum utilization in China.

| Authors | Publication year | Province | Methodology | Sample size | Data collection method | Results | Critical appraisal |
| --- | --- | --- | --- | --- | --- | --- | --- |
| Anson, O. | 2004 | Hebei | Cross-Sectional study | 4273 | Personal interview survey | Factors which have facilitated effect on PPC utilization: Younger age (P<0.001); Higher education (P<0.001); Fewer children (P<0.001); White collar occupation (P<0.01); Living with parents-in-law (P<0.01); Availability of MCH worker in the village (P<0.001); A shorter distance between the village and a township health center (P<0.05)/ county hospital (P<0.05)/ city hospital (P<0.01)  Factors which have no influence on PPC utilization: Per-capita income, living with parents, head of household, industry/agriculture occupation, village doctor commissioned for MCH. | 6/11  (AHRQ) |
| Chen, L., et al., | 2014 | Hebei | Mixed method study | 1601 | Online survey, semi-structured interviews | For mothers: A very small proportion (2.5%) of mothers reported they did not want visitors during the postnatal period. 65% of them said that they did not know about postnatal care and 24% of them thought it was unnecessary. Mothers mentioned accessibility factors (such as cost, and too long distance from home to health facility) as barriers for them to seek postnatal care services.  For health workers: Regarding the barriers to postnatal home visits, we found three subthemes from qualitative interviews: 1) understaffing, 2) inadequate in-service training, and 3) inconvenient transportation. | 15/17  (MMAT) |
| Fan, X., et al., | 2018 | Shaanxi | Cross-Sectional study | 2398 | National survey data | Factors which have facilitated effect on PPC utilization: Health reform in both urban (P=0.003) and rural (P=0.002); Give birth in a primary institution in urban area (P=0.011); Maternal education higher than primary school in rural area (P=0.008).  Factors which have no influence on PPC utilization: Maternal age, maternal education in urban area, employment, annual household income, parity, institution in rural area, way of giving birth, weight of newborn. | 7/11  (AHRQ) |
| Gu, H., et al. | 2018 | Jiangsu | Cross-Sectional study | 884 | Personal interview survey | Factors which have facilitated effect on PPC utilization: Higher education level (P<0.001); Shorter distance from the nearest hospital(<1km) (P<0.01); Higher income (P<0.01); Primiparous was negatively associated with nonuse of postpartum family visits (P<0.001). Deficient use (<3 visits of PPC) of postpartum family visits was positively related with longer distance (≥1km) (P<0.001); Give birth in high leveled institution (secondary and above) (P<0.01); Multiparous (P<0.05)  Factors which have inhibited effect on PPC utilization: Deficient use (<3 visits of PPC) of postpartum family visits was inhibited by employment (SES) (P<0.01).  Factors which have no influence on PPC utilization: Maternal age, marital status, education as influenced factor for deficient use of postpartum family visit; employed, hospital/institution, chronic disease, Way of giving birth. | 10/11  (AHRQ) |
| Gu, H., et al., | 2017 | Jiangsu | Cross-Sectional study | 946 | Personal interview survey | Factors which have inhibited effect on PPC utilization (≥3 visits): Internal migrants (P<0.001); Higher partner’s education level (P<0.001).  Factors which have facilitated effect on PPC utilization: Unemployment ((P<0.001); Obtain a medical insurance (P<0.001); Contravening the one-child policy (P<0.001).  Factors which have no influence on PPC utilization: Maternal age and education, annual household income per capita. | 8/11  (AHRQ) |
| Jiang, K., et al., | 2020 | Heilongjiang | Cross-Sectional study | 481 | Personal interview survey | PPC utilization (≥3 visits) was facilitated with natural birth (P<0.05); younger maternal age (<20 years old) (P<0.05).  Factors which have no influence on PPC utilization: Obtain a health insurance, ethnicity, maternal education level, child’s sex, times of childbearing and children’s number. | 9/11  (AHRQ) |
| Klemetti, R., et al., | 2011 | Chongqing; Shaanxi | Cross-Sectional study | 2576 | Personal interview survey | Unauthorized birth was negatively related with PPC utilization (P<0.001 for any postnatal visits and P=0.001 for three or more postnatal visits). This was only found in Shaanxi, not in Chongqing. | 9/11  (AHRQ) |
| Liu, X., et al., | 2014 | Gansu; Guangxi; Guizhou; Jiangxi; Inner Mongolia; Ningxia; Qinghai; Sichuan; Xinjiang; Chongqing | Cross-Sectional study | 14112 | Personal interview survey | Increasing SES was positively related with PPC utilization (≥2 visits) (includes women's age, ethnicity, and husband education, middle wealth index) (P<0.05).  Factors which have no influence on PPC utilization: Parity. | 6/11  (AHRQ) |
| Liu, X., et al., | 2011 | Gansu; Guangxi; Guizhou; Jiangxi; Inner Mongolia; Ningxia; Qinghai; Sichuan; Xinjiang; Chongqing | Cross-Sectional study | 14112 | Personal interview survey | higher household wealth index (Lowest OR in Xinjiang 0.38 vs Highest OR in Ningxia 2.60) and live in the county at lower altitude (OR=0.49 for altitude 501-1500m and OR=0.54 for altitude ≥1500m) was positively associated with higher MHC utilization. Sufficient prenatal visits (OR, 1.86; 95% CI, 1.58-2.19) and giving birth in hospitals (OR, 1.53; 95% CI, 1.23-1.90) were positively associated with postnatal visit.  Factors which have no influence on PPC utilization: Parity, ethnicity, family member, maternal age, maternal education, husband’s education. | 6/11  (AHRQ) |
| Liu, Y.H., et al., | 2006 | Hebei; Shanxi; Jiangsu; Zhejiang | Cross-Sectional study | 368589 | Survey | Higher PPC utilization was found in south China (92.2%~95.7% from 1994 to 2000). Urban area in north China has the lowest PPC utilization ratio (even lower than rural area in north China). | 7/11  (AHRQ) |
| Lomoro, O., et al., | 2002 | Shanghai | mixed method study | 50 | Semi-structured interviews | Postpartum women want high quality postpartum care; Time pressure of health workers; Lack of professional skills of health providers | 15/17  (MMAT) |
| Nan, Y., et al., | 2020 | Shaanxi | A descriptive phenomenological study (qualitative study) | 28 | Face-to-face interviews | Factors which were associated with lower PPC utilizations: dissatisfaction with current professional services; Insufficient communication time (low quality of service, dissatisfaction); insufficient willingness to seek professional help; too expensive; embarrassed to discuss her private issues; saving face culture/domestic shame; following confinement requirement and family burden. Mother preferred professional online platforms. | 9/10  (CASP) |
| Shen, Y., et al., | 2014 | Shaanxi | Cross-Sectional study | 2562 | Personal interview survey | Poor women were found to have less usage of ≥ 3 postnatal care services than non-poor women in 2008-2009 (P<0.05, CI = 0.0527). | 11/11  (AHRQ) |
| Shen, Y., et al., | 2019 | Shaanxi | RCT | 1113 | Personal interview survey | Training intervention was more effective for improving the PPC utilization than financial intervention (P<0.001). | 7/11  (AHRQ) |
| Tang, J., et al., | 2008 | Sichuan | Cross-Sectional study | 462 | Personal interview survey | PPC utilization ratio was facilitated with obtaining a medical insurance (P<0.05), higher maternal education (P<0.05), and higher family income (P<0.05).  Factors which have no influence on PPC utilization: Distance (>3km); different insurance types. | 6/11  (AHRQ) |
| Tang, M., et al., | 2015 | 31 provinces in mainland China | Cross-Sectional study | 7304 | National survey data | Factors which have inhibited effect on PPC utilization: Highest PPC utilization in the northwest (vs. lowest in the north China) (P<0.01); more prenatal examination (P<0.01); higher hospital giving birth rate (P<0.05); Urban area (vs. rural) (P<0.01). | 6/11  (AHRQ) |
| Tao, F., et al., | 2011 | Anhui | Mixed method study | 2326 | Focus group discussion, in-depth interview, personal interview survey | Inadequate government funds, understaffing, focusing on income generating services by care providers, and lack of awareness about postpartum care benefits were associated with lower PPV utilization ratio. | 14/17  (MMAT) |
| Wu, Y., et al., | 2015 | Sichuan | Cross-Sectional study | 284 | Personal interview survey | Factors which have inhibited effect on PPC utilization: Maternal age between 21-30 and 31-40 vs. ≤20 and >41 (P=0.004); Lower maternal education level (P=0.008); Less children (≤3) (P=0.027).  Factors which have no influence on PPC utilization: The number of family members, medical insurance, family income. | 9/11  (AHRQ) |
| Wu, Y., et al., | 2019 | Sichuan | Cross-Sectional study | 760 | Personal interview survey | Factors which have facilitated effect on PPC utilization: Younger age (P<0.01); Higher maternal education level (P<0.01); Han ethnicity (P<0.01); First child (P<0.01); Migrant worker (P<0.05); Possession of fixed family assets (P<0.01); Well-equipped township health centers (P<0.01); Good techniques in township health centers (P<0.05); Usage of antenatal care (P<0.01).  Factors which have no influence on PPC utilization: Whether township health centers/county hospitals can provide services, good doctors in township health centers/county hospital, good sanitation, well-equipped county hospital, good techniques in county hospital, travel time to hospital (<1h), higher maternal care knowledge score. | 6/11  (AHRQ) |
| Wu, Z., et al., | 2011 | China | Cross-Sectional study | 12171 | National survey, interview | urban area was more likely to receive a PPC service than rural area (P<0.01). Women in richest rural area were 1.81 more likely to receive postnatal care. | 11/11  (AHRQ) |
| Xiang, Y.X., et al., | 2014 | Zhejiang; Henan; Qinghai; Chongqing | Cross-Sectional study | 347 | Personal interview survey | Factors which have inhibited effect on PPC utilization (≥3 visits): Availability of home visiting (P=0,001); Service quality (P=0.008); preterm birth (P=0.030); Low birth weight (P=0.002).  Factors which have inhibited effect on PPC utilization (1-2 visits): Obtaining of a giving birth expenses compensation (P=0.002); Suburb village type (vs. rural village) (P=0.015); Women who considered the postpartum care services they received as high quality (P=0.014).  Factors which have no influence on PPC utilization: Maternal education, maternal employment, annual personal income, geographical divisions, birth method, preterm birth and low birth weight for 1-2 PPC visits, compensation for giving birth expenses and village type for standard PPC use (≥3 visits). | 8/11  (AHRQ) |
| You, H., et al., | 2016 | Zhejiang | Cross-Sectional study | 154 | Personal interview survey | NCMS policy improved the postnatal visit rate (P= 0.009). | 7/11  (AHRQ) |
| You, H., et al., | 2013 | Zhejiang | Cross-Sectional study | 223 | Personal interview survey | Factors which have inhibited effect on PPC utilization (≥3 visits): Compensation for giving birth fee expenses (P=0.02).  Factors which have inhibited effect on PPC utilization (1-2 visits): Maternal education (P=0.001); Annual household income (P=0.03); Parity (P<0.001); compensation for giving birth fee expenses (P=0.05).  Factors which have no influence on PPC utilization: Employment, the way of giving birth, premature labor, sex of neonates, birth weight of neonates. No influence was noticed from education, annual household income and parity on standard PPC utilization (≥3 visits). | 10/11  (AHRQ) |
| Yuan, Z., et al., | 2012 | Jiangxi | RCT | 10 townships and 58 villages | Personal interview survey | Health care intervention with CHS (community health services) model had a positive impact on the PPC services provided at village and township level (P=0.026). | 8/13 (JBI) |
| Zeng, L., et al., | 2008 | Shaanxi | Cross-Sectional study | 4794 | Personal interview survey | Families with lower income have higher PPC utilization rate for the free prenatal and postnatal visits in the poor regions in Shaanxi (P<0.001). | 6/11  (AHRQ) |
| Zhang, L., et al., | 2016 | Jiangsu | Cross-Sectional study | 988 | National survey | Factors which have facilitated effect on PPC utilization: younger husband’s age (P<0.05); Higher husband’s education (P<0.05); No previous pregnancy (P<0.05), Urban residence (P<0.01).  Factors which have no influence on PPC utilization: Whether living with parents, family per capita income, husband’s employment status, previous birth number, travel time.  Limitation: high risk of correlation between the variables (economic level, employment, or education), no conclusion of these influencing factors. | 10/11  (AHRQ) |
| Zhang, W., et al., | 2016 | Hunan | Cross-Sectional study | 1035 | Personal interview survey | Factors which have facilitated effect on PPC utilization: Older maternal age (>25) (P<0.05); Higher family per capita income (P<0.05); Primipara (P<0.05).  Factors which have no influence on PPC utilization: Maternal education, urban vs. rural area. | 10/11  (AHRQ) |
| Zhou, H., et al., | 2020 | Sichuan; Gansu | Quasi-experimental study design | 1522 | Survey | Conditional cash transfer has limited facilitated effect on postpartum care visit in partial eligible areas. | 7/9 (JBI) |
| Gong, S.Y., et al., | 2017 | 31 provinces in mainland China | Cross-Sectional study | 3270 | Personal interview survey | Factors which have facilitated effect on PPC utilization: Higher maternal education (P<0.05); Non-agriculture Hukou (P<0.05); Primipara (P<0.001).  Factors which have no influence on PPC utilization: Comply with birth policy, ethnicity. | 7/11  (AHRQ) |
| Han, S.Q., et al., | 2017 | Beijing; Qingdao; Zhejiang; Fujian; Guangdong | Cross-Sectional study | 1477 | National survey data | Factors which have facilitated effect on PPC utilization: Higher maternal education (P<0.05); Migrants within the same province (P<0.05).  Factors which have inhibited effect on PPC utilization: ≥3 children (P<0.01) | 7/11  (AHRQ) |
| Jiang, M.F, et al., | 2007 | Shanghai | Cross-Sectional study | 472 | Personal interview survey | Factors which have facilitated effect on PPC utilization:  Higher maternal education (P<0.05); Employed (P<0.05); Cost covered by employer (P<0.05); First child (P<0.05); Higher partner’s educational level (P<0.05); Partner’s medical cost was covered by insurance (P<0.05); Family income (P<0.05); Local hukou (P<0.05). | 5/11  (AHRQ) |
| Li, Z.X., et al., | 2020 | Sichuan | Cross-Sectional study | 620 | Personal interview survey | Obtain a maternity insurance was positively related with PPC utilization rate (≥3 times).  Factors which have no influence on PPC utilization: Maternal age, stay duration in current area, Hukou’s type, ethnicity, maternal and partner’s educational level, marital status, family income, miscarriage, past birth history, medical insurance, planned pregnancy, maternity health knowledge. | 8/11  (AHRQ) |
| Liu, Y.T., et al., | 2006 | Beijing; Shanghai; Guangdong; Zhejiang | Cross-Sectional study | 3532 | Personal interview survey | Factors which have facilitated effect on PPC utilization: Higher maternal educational level (P=0.000); Higher partner’s educational level (P=0.000); Partner has a medical insurance (P=0.000); Higher family income (P=0.000).  Factors which have no influence on PPC utilization: Maternal age, maternal and partner’s employment status, mother has a medical insurance. | 6/11  (AHRQ) |
| Ning, L.X., et al., | 2014 | Jiangxi | Cross-Sectional study | 546 | National survey data | The PPC utilization rate was higher in urban than in rural (P<0.001). | 5/11  (AHRQ) |
| Pan, J.Q., et al., | 2018 | Kunshan | Cross-Sectional study | 536 | Online survey | Factors which have facilitated effect on PPC utilization: Local Hukou (P<0.01); Higher maternal educational level (P<0.01); Younger maternal age (P<0.05); Maternal educational level under high school (P<0.05); Urban taxpayers (P<0.05)  Factors which have inhibited effect on PPC utilization: Maternal education level was junior high school, distance to the nearest hospital, employment, income. | 9/11  (AHRQ) |
| Song, A.H., et al., | 2007 | Chongqing | Qualitative study | 93 | Focus group discussion; in-depth interviews | Inadequate governmental investment in maternal and child health care caused resulting in unguaranteed salaries for health care workers, understaffing, lack of professional skills of health providers. | 6/10  (CASP) |
| Tian, Y., et al., | 2013 | Shanghai | Cross-Sectional study | 450 | Personal interview survey | Factors which have facilitated effect on PPC utilization: Local Hukou (P=0.002); Higher maternal educational level (P=0.023). | 9/11  (AHRQ) |
| Wang, Y.Y., et al., | 2016 | Guangzhou; Zhejiang; Fujian; Beijing | Cross-Sectional study | 346 | National survey data | Factors which have facilitated effect on PPC utilization: Higher maternal education level (higher than secondary school) (P=0.040); Lower family expenses (<4000RMB) (P=0.047); Received health education in local institutions (P=0.000).  Factors which have no influence on PPC utilization: Maternal age, birth policy, first child, belongs to certain local organization, relationship with local people. | 6/11  (AHRQ) |
| Yang, Q., et al., | 2014 | Shanghai | Cross-Sectional study | 604 | Survey | Local Hukou was positively related with PPC utilization rate (P<0.001). | 8/11  (AHRQ) |
| Yu, Z.J., et al., | 2013 | Shandong; Henan; Gansu | Cross-Sectional study | 2021 | Personal interview survey | Factors which have facilitated effect on PPC utilization: Urban (vs. rural) (P=0.000); Higher maternal educational level (P=0.000); Higher family income (P=0.001 for postpartum consultation, P=0.042 for day 7 postpartum visit, P=0.000 for day 42 postpartum visit). | 7/11  (AHRQ) |
| Zhang, J.R., et al., | 2017 | 31 provinces in mainland China | Cross-Sectional study | 4838 | National survey data | Factors which have facilitated effect on PPC utilization:  Higher maternal age (P<0.001); Higher partner’s educational level (P<0.01~0.003 for different educational levels); geographical allocation (Higher PPC in eastern China, P=0.005); Employed (P=0.012); Higher income (P=0.03~0.007 for different income groups), More children (P<0.001). | 6/11  (AHRQ) |

AHRQ: Agency for Healthcare Research and Quality checklist. Article quality is assessed as follows: low quality=0-3; moderate quality=4-7; high quality=8-11

CASP: Critical Appraisal Skills Programme Qualitative Research Checklist

MMAT: Mixed Methods Appraisal Tool

JBI: Joanna Briggs Institute Critical Appraisal tools

Table A. Search strings for both English and Chinese databases

| Databases | Search strings |
| --- | --- |
| Medline | ("china"[MeSH Terms] OR "china"[Title/Abstract] OR "Chinese"[Title/Abstract]) AND ("utili*"[Title/Abstract] OR "use"[Title/Abstract]) AND ("Postpartum Period"[MeSH Terms:noexp] OR "Postpartum"[Title/Abstract] OR "Post-partum"[Title/Abstract] OR "Post-Natal"[Title/Abstract] OR "Postnatal"[Title/Abstract] OR "puerper*"[Title/Abstract] OR "after birth"[Title/Abstract] OR "after delivery"[Title/Abstract]) |
| Web of science (core collection) | TS=("china" OR "Chinese") AND  TS=("Utili*" ) AND TS=("Postpartum" OR "Post-partum" OR "Post-Natal" OR "Postnatal" OR "Puerper*" OR "after birth" OR "after delivery") |
| Embase | ('china'/exp OR china:ti,ab,kw,ad,ff OR chinese:ti,ab,kw,ad,ff) AND ('care'/exp OR consultation:ti,ab,kw,ad,ff OR (home:ti,ab,kw,ad,ff AND visit:ti,ab,kw,ad,ff) OR service:ti,ab,kw,ad,ff OR plan:ti,ab,kw,ad,ff OR care) AND ('utilization'/exp OR utili*:ti,ab,kw,ad,ff) AND ('puerperium'/exp OR 'puerperium':ti,ab,kw OR 'postpartum period':ti,ab,kw OR 'postpartum':ti,ab,kw OR 'post-partum':ti,ab,kw OR 'post-natal':ti,ab,kw OR 'postnatal':ti,ab,kw OR 'puerper*':ti,ab,kw OR 'after birth':ti,ab,kw OR 'after delivery':ti,ab,kw) |
| Wan Fang | (主题:(产褥期) or主题: (产后)) and (主题:(服务利用)) |
| China National Knowledge Infrastructure (CNKI) | (TKA=产褥期 OR TKA=产后) AND (TKA=服务利用 OR TKA=影响因素) |
